# Dengue Fever Culminating in Cardiac Arrest: A Case Report

**DOI:** 10.7759/cureus.61063

**Published:** 2024-05-25

**Authors:** Fawaz Mohammed, Matthew McCurdy, Jeffrey Foley, Zaina Ali Khan, Shamaila Mohammed, Mallory Vaughn, Samantha Ruley, Matthew D. Stone, Akhtar Amin, Jacqueline Dawson Dowe, Mohammed Kazimuddin, Muhammad Akbar, Sameer Saleem

**Affiliations:** 1 Internal Medicine, University of Kentucky College of Medicine, Bowling Green, USA; 2 Cardiology, University of Kentucky College of Medicine, Bowling Green, USA; 3 College of Medicine, Deccan College of Medical Sciences, Hyderabad, IND; 4 General Medicine, Bhaskar Medical College, Hyderabad, IND; 5 Cardiology, Medical Center Bowling Green, Bowling Green, USA

**Keywords:** temporary pacemaker, travel-related infection, cardiac arrest, third-degree heart block, dengue fever/complications

## Abstract

Infection from the dengue virus can manifest with a variety of clinical presentations. Cardiac involvement from dengue fever is a rarely reported phenomenon with significant morbidity and mortality. We illustrate the case of a 47-year-old male admitted to the hospital with fevers. The hospital course was complicated with cardiac arrest. Clinicians need to be weary of this rare occurrence particularly in areas with a known prevalence of dengue for prompt recognition and improved patient outcomes.

## Introduction

Transmitted by the *Aedes aegypti* mosquito, dengue is a single-stranded RNA virus belonging to the *Flaviviridae* family. Infection from dengue can have various clinical manifestations ranging from a mild fever to lethal dengue hemorrhagic fever or dengue shock syndrome from diffuse capillary leakage [1]. A small portion of individuals presenting with organ involvement have expanded dengue syndrome where the disease can manifest in the heart, liver, or nervous system [2,3]. Cardiac involvement can have variable presentations in the form of myopericarditis, atrioventricular conduction abnormalities, or non-specific electrocardiogram changes [4]. Cases of myocarditis have been reported in the literature, but complete atrioventricular blocks have rarely been described [5]. Herein, we report a rare occurrence of cardiac arrest in the setting of dengue fever in an individual who presented to the hospital after returning from Cuba.

## Case presentation

**Figure 1:**
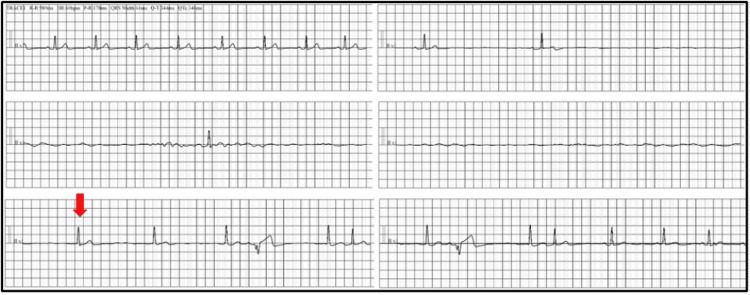
Telemetry series Telemetry series demonstrating normal sinus rhythm followed by unprovoked asystole event leading to cardiogenic syncope. Eventual return of junctional escape rhythm (arrow) with ventricular premature complexes and atrial premature complexes

**Figure 2:**
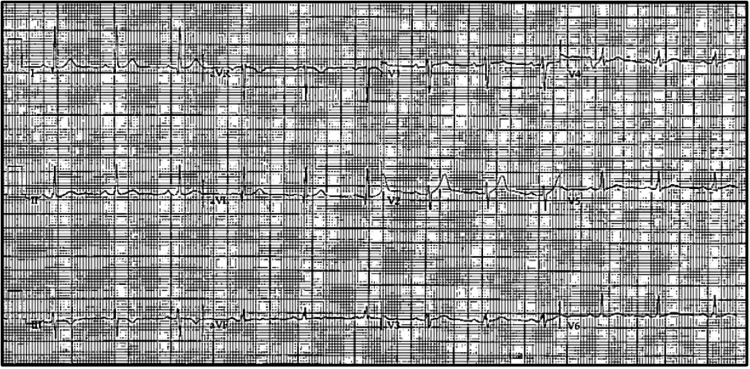
Electrocardiogram post return of spontaneous circulation Electrocardiogram obtained immediately following cardiac arrest demonstrating normal sinus rhythm without other significant findings

**Figure 3:**
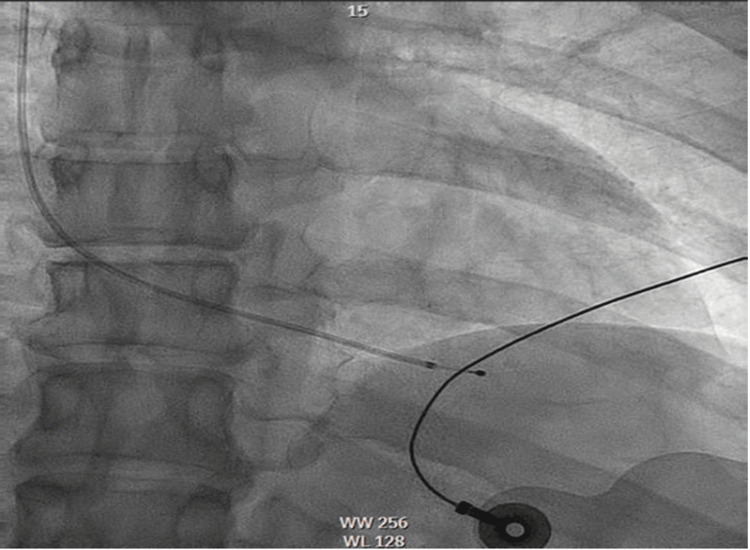
Right heart catheterization with the placement of a temporary transvenous pacemaker

**Figure 4:**
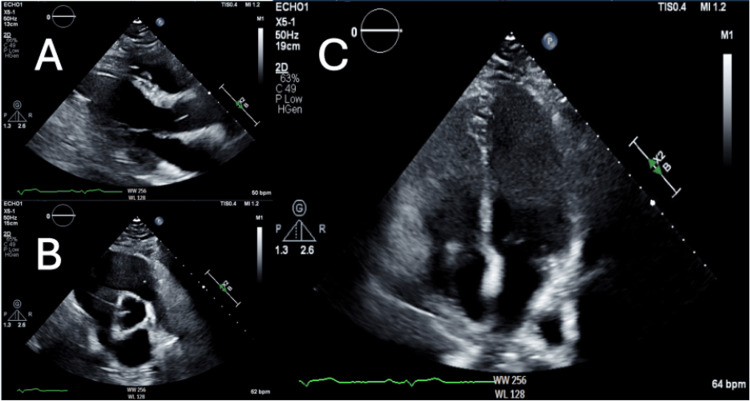
Transthoracic echocardiogram (A) Transthoracic echocardiogram, long axis, demonstrating normal wall thickness and aortic root. (B) Basal short axis view demonstrating normal aortic root. (C) Apical four-chamber view demonstrating normal chamber sizes

A 47-year-old Hispanic male with recent travel to Cuba presented to the emergency department (ED) with complaints of fevers, nausea, vomiting, and diarrhea of one-week duration. He had a past medical history of hyperlipidemia. On presentation, he had reported that several members of his family were sick when he had visited Cuba attributed to be secondary to dengue fever. Upon presentation to the ED, he was noted to be febrile with a temperature of 101.8°F, a heart rate of 66/min, a respiratory rate of 18/min, and a blood pressure of 138/84 mmHg. Physical examination was not significant. Labs on admission showed leukopenia at a count of ­­­­­­­­2.6 (4-11×10^9^/L), hemoglobin 14.6 g/dL (13-18 g/dL in males), hematocrit 43.5% (35-45%), thrombocytopenia 61.0×10^9^/L (150-400×10^9^/L), prothrombin time 14.1 seconds (sec) (11.6-14.9 sec), and activated partial thromboplastin time 40.0 sec (25-35 sec). Liver function tests revealed elevated levels of aspartate aminotransferase to 111 IU/L (<40 IU/L), alanine transaminase to 79 IU/L (<40 IU/L), and alkaline phosphatase to 67 IU/L (<150 IU/L) and decreased albumin levels at 3.3 g/dL (>4 g/dL). Electrocardiogram (EKG) on presentation was normal with no ST-T wave abnormalities, and troponins were within normal limits. On hospital day 2, the patient had complained of lightheadedness on evaluation. He then sat at the edge of the bed and subsequently fell and lost consciousness. He then gained consciousness spontaneously. A CT of the head was obtained which showed no intracranial abnormalities. Cardiology was consulted, and he was transferred to the cardiac care unit for telemetry monitoring. Later that morning, he became unresponsive with no palpable pulse. Code blue was activated; however, within 10 seconds, return of spontaneous circulation (ROSC) was achieved. Telemetry strips were reviewed, and they demonstrated normal sinus rhythm followed by asystole (Figure 1). Post spontaneous ROSC, his EKG demonstrated no evidence of ST changes suggestive of acute ischemia or pre-excitation (Figure 2). A decision was made by cardiology to place a temporary transvenous pacemaker given his hemodynamic compromise from bradycardia (Figure 3). On hospital day 3, infectious disease was consulted due to his ongoing fevers complicated with bradycardia. Given his diarrhea and recent travel history, there was initial suspicion of *Salmonella typhi* as the etiology of bradycardia through sphygmothermic dissociation (bradycardia with fever). He was empirically initiated on ceftriaxone; however, *Salmonella typhi* infection was ruled out with a negative gastrointestinal polymerase chain reaction (PCR). Serology was positive for dengue PCR type 3 and dengue virus-specific IgG consistent with a diagnosis of dengue fever. A transthoracic echocardiogram was obtained and showed a left ventricular ejection fraction (LVEF) of 55-60% with normal left ventricle wall motion. The right ventricle was mildly dilated. There was no evidence of pericardial effusion (Figure 4). Antibiotics were discontinued and he was managed supportively. On hospital day 7, his transvenous temporary pacemaker was removed without complications. Monitoring on telemetry showed no recurrence of bradycardia and atrioventricular nodal conduction abnormalities. Electrophysiology recommended no permanent pacemaker placement, and he was discharged with a Holter monitor and set to follow up with cardiology as an outpatient.

## Discussion

Close to 100 million cases of dengue have been reported annually making it the most prevalent mosquito-borne illness across the globe [6]. The disease itself progresses through three stages: (i) febrile phase with flu-like symptoms, (ii) critical phase having pronounced capillary leakage, and (iii) convalescent phase [7]. The virus has a predilection for cardiac tissue, leading to increased morbidity and mortality from the disease. The DENV-2 and DENV-3 serotypes have been typically described to have cardiac involvement [8]. This may be due to the direct effect of the virus on the cardiac muscle or indirectly from the activation of an immunological cascade or both processes occurring in conjunction [8]. Complications arising from cardiac involvement can be numerous but rarely have life-threatening consequences. Most commonly, arrhythmias are seen as a complication with sinus tachycardia being the most reported. First-degree and second-degree heart blocks have also been more frequently reported than complete heart block. From a pathophysiological perspective, myocarditis from the virus occurs due to viral tropism for the heart with the resultant host immune response leading to cytokine storm causing damage to the heart musculature [9]. Functional abnormalities from altered automaticity and adenosine metabolism can lead to conduction abnormalities [10]. Conduction abnormalities with multiple electrolyte derangements are commonly seen with dengue. Modalities in the form of cardiovascular magnetic resonance (CMR) and endomyocardial biopsy can help with diagnosis; however, their use may be of limited value. When myocarditis is suspected, elevated serological markers of cardiac injury along with EKG and echocardiographic changes can help identify myocarditis [11]. Management largely depends on the level of hemodynamic compromise and relies on the prompt recognition of cardiac abnormalities with subsequent supportive management. Myocarditis is generally transient resolving spontaneously with the restoration of hemodynamic parameters although rarely a pacemaker may be required.

## Conclusions

Most cases of cardiac complications from dengue fever are self-limiting with a minority of cases progressing requiring close monitoring. Prompt recognition of dengue-related cardiac complications is important for better outcomes. There is a need for further research for more definitive management protocols to allow for the optimum treatment of individuals presenting with cardiac complications from dengue fever. 

## References

[1] Teixeira MG, Barreto ML (2009). Diagnosis and management of dengue. BMJ.

[2] Arif A, Abdul Razzaque MR, Kogut LM, Tebha SS, Shahid F, Essar MY (2022). Expanded dengue syndrome presented with rhabdomyolysis, compartment syndrome, and acute kidney injury: a case report. Medicine (Baltimore).

[3] Guzman MG, Harris E (2015). Dengue. Lancet.

[4] Yacoub S, Wertheim H, Simmons CP, Screaton G, Wills B (2014). Cardiovascular manifestations of the emerging dengue pandemic. Nat Rev Cardiol.

[5] Virk HU, Inayat F, Rahman ZU (2016). Complete heart block in association with dengue hemorrhagic fever. Korean Circ J.

[6] Bhatt S, Gething PW, Brady OJ (2013). The global distribution and burden of dengue. Nature.

[7] (2009). Dengue: guidelines for diagnosis, treatment, prevention and control.

[8] Hober D, Delannoy AS, Benyoucef S, De Groote D, Wattré P (1996). High levels of sTNFR p75 and TNF alpha in dengue-infected patients. Microbiol Immunol.

[9] Tisoncik JR, Korth MJ, Simmons CP, Farrar J, Martin TR, Katze MG (2012). Into the eye of the cytokine storm. Microbiol Mol Biol Rev.

[10] Sharma JK, Zaheer S (2014). Variable atrio-ventricular block in dengue fever. J Indian Acad Clin Med.

[11] Caforio AL, Pankuweit S, Arbustini E (2013). Current state of knowledge on aetiology, diagnosis, management, and therapy of myocarditis: a position statement of the European Society of Cardiology Working Group on Myocardial and Pericardial Diseases. Eur Heart J.

